# Progress in combination vaccines and the co-administration of influenza virus and SARS-CoV-2 vaccines

**DOI:** 10.3389/fimmu.2025.1578733

**Published:** 2025-06-25

**Authors:** Chengyu Hu, Chenguang Niu, Xiaohui Li, Ke He, Mengyu Li, Xiaonan Gao, Qiannan Wei, Weiyang Sun, Yongkun Zhao, Yuanguo Li, Xianzhu Xia, Zhiguang Ren, Xiaodong Li, Tiecheng Wang

**Affiliations:** ^1^ Henan Province Engineering Technology Research Center of Intelligent Diagnosis and Treatment, The First Affiliated Hospital, School of Basic Medicine Sciences, Henan University, Kaifeng, China; ^2^ Institute of Traditional Chinese Medicine, School of Pharmacy, Henan University, Kaifeng, China; ^3^ Changchun Veterinary Research Institute, Chinese Academy of Agricultural Sciences, Changchun, China; ^4^ Jiangsu Co-innovation Center for Prevention and Control of Important Animal Infectious Diseases and Zoonoses, Yangzhou University, Yangzhou, China

**Keywords:** SARS-CoV-2, influenza virus, coinfection, coadministration, combination vaccines

## Abstract

COVID-19 and seasonal influenza have taken a huge toll on the global economy and global health. Given the potential of COVID-19 to transform into a chronic epidemic akin to seasonal influenza, the influenza virus and SARS-CoV-2 will continue to be a significant threat to healthcare for some time to come. Coinfection involving the two viruses has been proven to worsen the severity of the illness, as evidenced by clinical observational data. Vaccination remains the most effective measure in the prevention and treatment of infectious diseases. In addition, the coadministration of influenza virus and SARS-CoV-2 vaccines offered greater benefits than either vaccine alone. Combination vaccines are also a major hotspot in novel vaccine development. This review highlights the advancements in the development of combined vaccines for COVID-19 and seasonal influenza, as demonstrated in animal studies and clinical trials, and emphasizes the importance of a combined vaccine.

## Graphical Abstract

Confirmed COVID-19 and influenza cases.

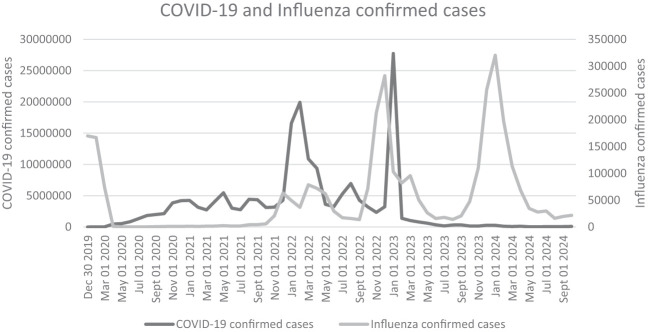

## 1 Introduction

In the past few years, the annual seasonal influenza outbreaks and the worldwide COVID-19 pandemic have inflicted substantial harm on the global economy and human health (1, 2). Despite both being respiratory viruses that primarily manifest as respiratory symptoms, such as nasal discharge, sore throat, cough, and headache, among others, there exist numerous distinctions between SARS-CoV-2 and the influenza virus (3–12). Initially, it was hypothesized that SARS-CoV-2 would supplant influenza viruses as it spread worldwide (13). However, subsequent pandemics have revealed the emergence of novel virus-virus interactions, wherein the co-circulation of influenza viruses and SARS-CoV-2 strains has been reported (14). The coinfection of SARS-CoV-2 with the influenza virus has been found to exacerbate the severity of disease manifestation in humans (15), but the implementation of maintaining social distancing measures appeared to mitigate the impact of such co-infections (16). However, the relaxation of public gathering restrictions and travel limitations was likely to result in a significant increase in co-infection involving the influenza virus.

**Figure 1:**
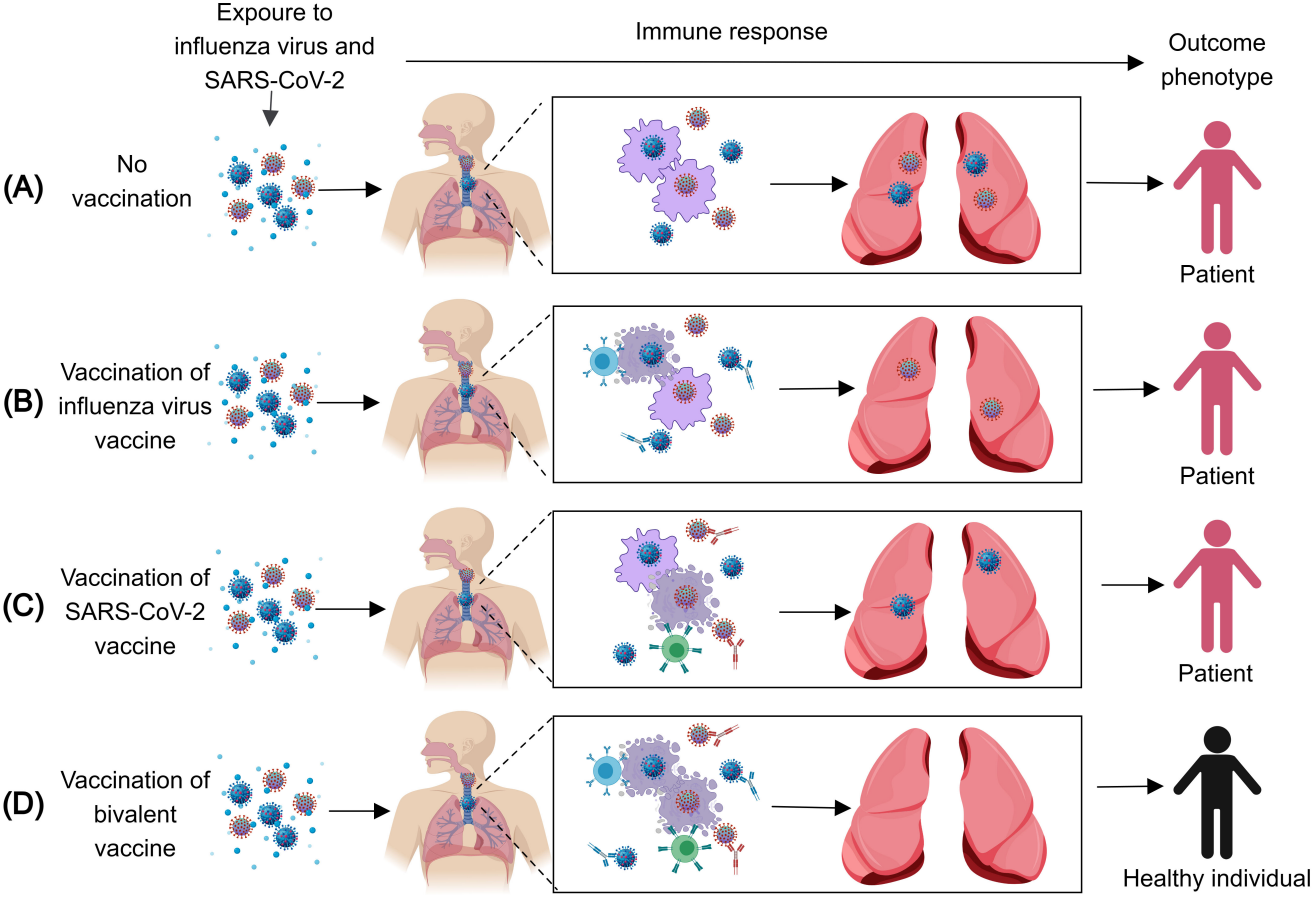
The immune response after infection with the influenza virus and SARS-CoV-2. **(A)** When exposed to the influenza virus and SARS-CoV-2, unvaccinated Individuals cannot produce the corresponding antibodies and effector T cells against the influenza virus and SARS-CoV-2, thus, they will be infected with the influenza virus and SARS-CoV-2. **(B)** When exposed to the influenza virus and SARS-CoV-2, individuals vaccinated with the influenza virus vaccine can produce the corresponding antibodies and effector T cells against the influenza virus, thus, they will be infected with SARS-CoV-2. **(C)** When exposed to the influenza virus and SARS-CoV-2, individuals vaccinated with the SARS-CoV-2 vaccine can produce the corresponding antibodies and effector T cells against SARS-CoV-2, thus, they will be infected with the influenza virus. **(D)** When exposed to the influenza virus and SARS-CoV-2, individuals vaccinated with the influenza virus vaccine and SARS-CoV-2 vaccine can produce the corresponding antibodies and effector T cells against the influenza virus and SARS-CoV-2, thus, the individuals cannot be infected by either virus.

A reduction in the occurrence of SARS-CoV-2 infection has been noted with influenza vaccination (17). Furthermore, the combination of influenza and SARS-CoV-2 vaccinations has been validated to have an enhanced immunizing effect against SARS-CoV-2 infection, hospitalizations, and overall mortality (18). Clinical trials have additionally confirmed the feasibility of simultaneous vaccination as an effective immunization approach (18). Consequently, the development of combined vaccines targeting both SARS-CoV-2 and influenza may present a more advantageous alternative. Nearly 30 different combination vaccines for SARS-CoV-2 and the influenza virus have been documented, including recombinant live attenuated virus vaccines, mRNA-based vaccines, recombinant protein vaccines, and virus-like particle (VLP) vaccines. All the combination vaccines that have been proven in animal studies could produce robust neutralizing antibodies and protect from both SARS-CoV-2 and influenza infection (**Figure 1**) (1). Some combination vaccines are already in clinical trials. The good news is that one clinical trial on a nasally administered combination vaccine against influenza and COVID-19, based on a live-attenuated influenza virus vector, has been shown to be tolerable in adults. Here, we review the characteristics and research progress of these combination vaccines.

## 2 Influenza virus and SARS-CoV-2

### 2.1 Influenza virus

According to the World Health Organization (WHO), worldwide, annual epidemics of influenza are estimated to result in approximately 3 to 5 million cases of severe illness and about 290,000 to 650,000 respiratory deaths (2). All age groups are affected, but some groups are more at risk than others, as people at greater risk of severe disease or complications when infected are pregnant women, children under 59 months, the elderly, individuals with chronic medical conditions (such as established cardiac, pulmonary, renal, metabolic, neurodevelopmental, liver, or hematologic diseases), and individuals with immunosuppressive conditions (such as HIV/AIDS, receiving chemotherapy or steroids, or malignancy) (2). In industrialized countries, most influenza-related deaths occur among people aged 65 or older (3). The adoption of measures fighting against the threat posed by COVID-19 since the first months of 2020 produced a remarkable decrease in the severity of seasonal influenza-like illnesses during the 2020/21 season (4–6). The COVID-19 control measures helped to prevent influenza from circulating during the 2020/21 season (7). The decrease in the severity of influenza-like illnesses may imply a lower population immunity for the season 2021/22 since most people had not been exposed to the influenza virus for over a year (8, 9). In the past 2 years, there was a more severe influenza outbreak compared to the pre-COVID-19 pandemic period (10).

Influenza viruses are categorized into types A, B, C, and D based on their core proteins, with only types A and B being associated with human diseases of concern. Type A viruses are further subdivided according to their hemagglutinin (HA) and neuraminidase (NA) proteins (11, 12). Despite the availability of effective antiviral drugs, vaccination remains a crucial preventive strategy. Since most of the neutralizing antibody targets are in the HA globular head domain, this region is an essential target for conventional influenza vaccines (13, 14). However, the high mutability of this domain facilitates antigenic drift (15), resulting in variable vaccine effectiveness (VE) ranging from 10% to 60% over the past decade, depending on the match between the vaccine and circulating strains (16). Creating a universal influenza vaccine is a major scientific priority. The vaccine needs to improve the scope and durability of protection against seasonal influenza. Furthermore, it should safeguard against pandemic strains (17, 18). Current research focuses on three key strategies to overcome existing limitations: targeting conserved viral epitopes, developing novel vaccine platforms, and utilizing advanced adjuvants (19). Promising conserved targets for universal vaccines include the HA stem domain (20, 21), Matrix Protein 2 Ectodomain (M2e) (22, 23), NP (24, 25), and NA (26, 27). These conserved epitopes, central to universal vaccine development, have shown enhanced immunogenicity and protection when delivered through innovative platforms (28). For instance, VLP vaccines mimic native viral structures to induce robust immune responses (29–32), nanoparticle vaccines present high-density antigen arrays with intrinsic adjuvant properties (33–35), viral vector vaccines enable mucosal delivery for local immunity (36, 37), and nucleic acid vaccines offer rapid adaptability to emerging outbreaks (38, 39). In conclusion, the development of broadly protective influenza vaccines remains an urgent unmet need in global health.

### 2.2 SARS-COV-2

As of 10 November 2024, the WHO had documented over 776 million confirmed COVID-19 cases and 7 million deaths worldwide (40). However, the reported COVID-19 data were likely undercounted compared to actual cases due to various reasons (41). In addition to acute symptoms including fever, headache, and cough, patients may develop Long COVID (Post-Acute Sequelae of SARS-CoV-2 infection), a condition characterized by persistent multisystem symptoms lasting weeks to months after initial infection (42, 43). Given their multisystem involvement and established association with psychosocial sequelae, these persistent symptoms can lead to substantial long-term morbidity (44). For two years, people endured the impact of COVID-19 (45). As more and more regions stopped testing for COVID-19, it appeared as if the disease had faded away. While the WHO (40) and Chinese Center for Disease Control and Prevention (CCDC) (46) surveillance data demonstrate a sustained decline in confirmed cases since January 2023, and Wang et al.’s clinical study found predominantly mild acute disease in hospitalized Omicron-infected patients (47), emerging evidence suggests significant life-altering consequences among Long COVID patients following SARS-CoV-2 infection (48).

SARS-CoV-2 is a type of coronavirus, similar to SARS and MERS (49). The virus particle surface is covered with spike proteins (S protein), which are arranged as trimers. During viral entry, the receptor-binding domain (RBD) located in the S1 subunit of the S protein specifically interacts with receptors [angiotensin-converting enzyme (ACE2)] on human cells (50); then, the S2 subunit of the S protein facilitates the fusion between the virus and the cell (51, 52). The RBD’s role in stimulating protective antibodies during immune defense highlights the spike protein’s importance as an antigen in vaccine development (53).

Demonstrated to cause severe multisystem disease in human populations (40, 54), SARS-CoV-2 exhibits high transmissibility via respiratory droplets and aerosols, facilitating its rapid global dissemination (55). Similar to influenza prevention strategies, vaccination remains a cornerstone of COVID-19 pandemic control measures (56). In vaccine target design, while the M protein (57) and NSP3, NSP4, and NSP6 (58, 59) demonstrate immunogenicity, the S and N proteins remain the predominant immunodominant epitopes (60, 61). As of 25 November 2024, global vaccine development efforts included 183 candidates in clinical trials and 199 in preclinical studies, spanning multiple platform technologies: inactivated vaccines, nucleic acid (RNA/DNA) vaccines, viral vector vaccines, VLP vaccines, and protein subunit vaccines. Several of these have received WHO emergency use authorization (62). Compared to conventional vaccine approaches, modern molecular-based platforms demonstrate distinct advantages for addressing both emerging and established viral threats, such as recombinant viral vector-based vaccines (63, 64), VLP-based vaccines (65–69), recombinant protein-based vaccines (70, 71), and nucleic acid-based technologies (72, 73). These innovative platforms enable more adaptable, scalable, and cost-effective vaccine development while maintaining rapid production timelines. Collectively, these technological advances have played a pivotal role in COVID-19 pandemic control efforts.

### 2.3 Coinfection of SARS-CoV-2 and influenza virus

**Figure 2:**
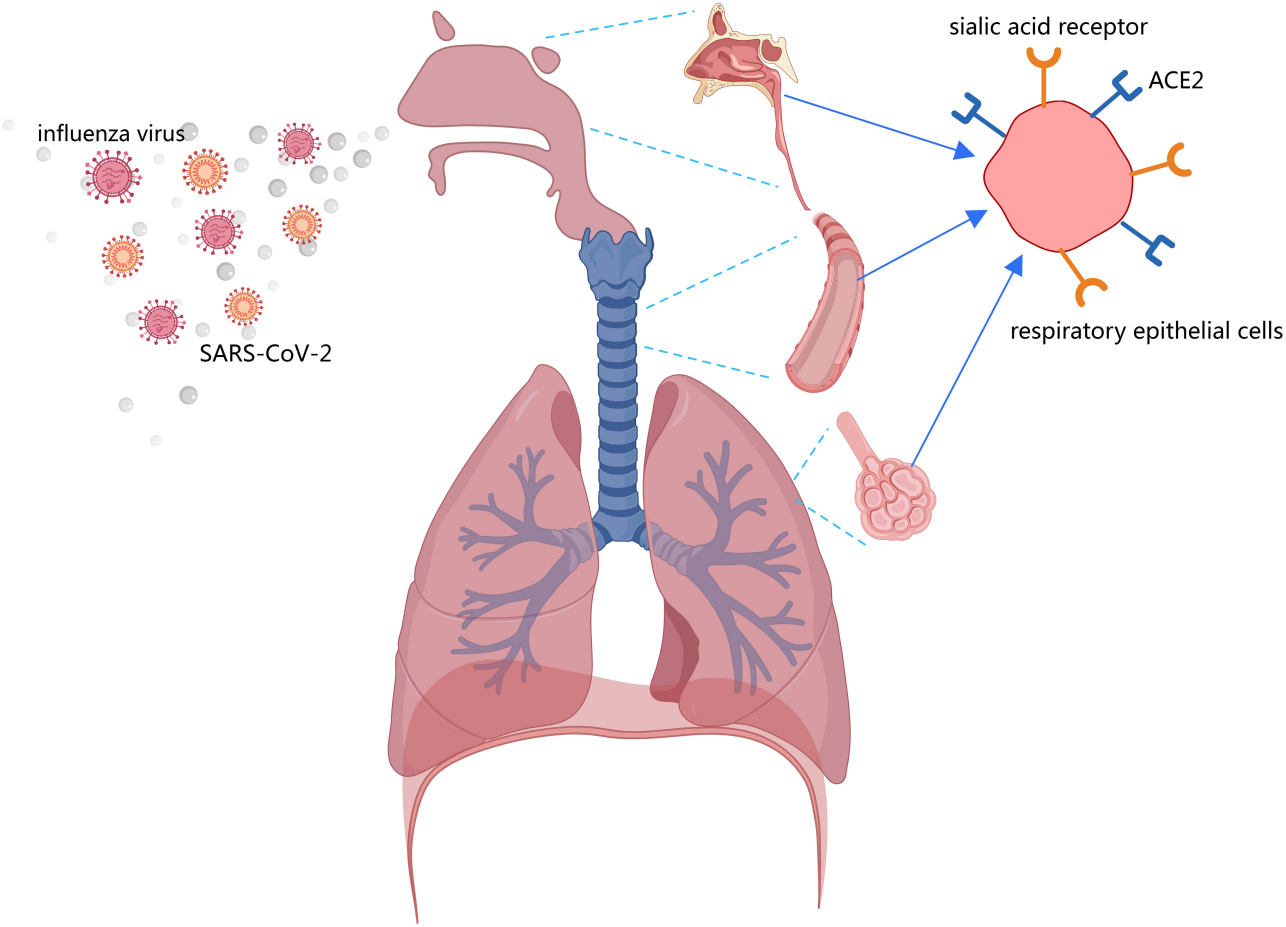
Both the sialic acid receptor (influenza virus receptor) and ACE2 are present in respiratory epithelial cells, including the nasal mucosa, trachea, bronchi, and alveoli.

As airborne pathogens, both the influenza virus and SARS-CoV-2 primarily target the human respiratory system, including the nasal mucosa, trachea, bronchi, and alveoli (74, 75). Effective preventive measures such as washing hands, social distancing, and mask-wearing can significantly reduce transmission risks for these pathogens (76). Notably, public health interventions implemented during COVID-19 outbreaks not only controlled SARS-CoV-2 spread but also suppressed seasonal influenza epidemics (77). However, following the relaxation of COVID-19 containment measures, influenza A virus (IAV) circulation rebounded to pre-pandemic levels, and in some cases exceeded them, while SARS-CoV-2 continued to circulate endemically (Graph 1) (78–80). Coinfection with IAV and SARS-CoV-2 has been reported in many regions (81–83), which may be facilitated by the co-localization of ACE2 and sialic acid receptors in respiratory tissues (**Figure 2**) (74, 75). While social distancing measures initially reduced coinfection risks (84), their subsequent removal, particularly travel restrictions, led to a marked increase in coinfection rates (85, 86). Importantly, pre-existing IAV infection had been shown to enhance SARS-CoV-2 infectivity (87), and clinical studies confirm that coinfected patients experience more severe disease manifestations (88, 89). A systematic review by Xiang et al. (2023) concluded that IAV-SARS-CoV-2 coinfection leads to worse clinical outcomes (90). Supporting this, Hashemi et al. (2023) reported a striking 22.3% coinfection rate with H1N1 influenza and SARS-CoV-2 among deceased patients in northeastern Iran (82). Collectively, these findings suggest that viral coinfections may contribute to elevated morbidity and mortality in patients with acute respiratory infections.

Furthermore, animal studies demonstrated that coinfection with the influenza virus and SARS-CoV-2 also resulted in more severe disease outcomes (91–93). In murine models, simultaneous infection with SARS-CoV-2 and H1N1 (compared to single-virus infection) prolonged the acute viral phase, exacerbated immune cell infiltration, and elevated inflammatory cytokine levels in bronchoalveolar lavage fluid, ultimately causing more severe pneumonia and pulmonary damage (93). Similar findings were observed in ferret models, where coinfected animals showed significantly greater weight loss and more pronounced inflammatory pathology in both upper (nasal turbinate) and lower (lung) respiratory tracts compared to single-virus infections (92). Most critically, Bao et al. (2021) reported that H1N1/SARS-CoV-2 coinfection in mice not only extended symptom duration and worsened pulmonary lesions but also resulted in significantly higher mortality rates than either monoinfection (91).

To confirm the coinfection rate of influenza and COVID-19, we analyzed all meta-analyses associated with coinfection of influenza and COVID-19 from PubMed up to 25 November 2024 (**Table 1**). The inaugural review in this field (94), which evaluated coinfection prevalence among SARS-CoV-2-confirmed patients, reported an overall viral coinfection rate of 3% (including influenza and other respiratory viruses). As shown in **Table 1**, the majority of the meta-analyses demonstrated that influenza coinfection was significantly correlated with worse clinical outcomes, particularly increased risks of ICU admission, mechanical ventilation requirement, and mortality (95–100). However, regional variations in disease severity and fatality rates were observed, likely attributable to differences in local public health policies (101). These findings were further corroborated by a large-scale UK study (n=7,000) that, through multivariable regression analysis, identified influenza coinfection as an independent risk factor for invasive mechanical ventilation in COVID-19 patients (102). During the peak COVID-19 pandemic period, reported coinfection rates ranged from 0.6% to 2.6% globally (95, 98, 99), potentially reflecting the combined effects of international containment measures (5, 7) and virus interference (103).

**Table 1:** Meta‐analysis of coinfection studies.

Time	Study purpose	Identified number of studies	Patients	Patient number	Influenza coinfection rate	Disease outcomes	Reference
1 January 1 to 17 April 2020	To evaluate the burden of coinfections in patients with COVID-19	30	All patients with simultaneous SARS-CoV-2 infection	3834	3% (including other viruses)	Cannot be evaluated	No CRD Lansbury et al., (2020) (94)
Up to 15 July 2021	To determine the proportion of coinfection with influenza viruses in SARS-CoV-2-positive patients	54	SARS-CoV-2 patients only report influenza virus infection	18021	2.2% (critically ill patients)	The proportion of coinfection with influenza viruses among critically ill patients was higher than that among patients overall.	No CRD Dao et al. (2021) (95)
Up to 9 July 2021	To determine the impact of coinfection with SARS-CoV-2 and influenza	12	COVID-19 patients received diagnostic testing for the influenza virus	11674	Cannot be evaluated	Mortality was significantly decreased in the studies from China and significantly increased outside China. There was a lower risk for critical outcomes among coinfection patients.	CRD42021267039 Guan et al. (2021) (101)
1 October 2019 to 8 February 2021	To examine the occurrence of coinfections and their outcomes among patients with SARS-CoV-2 infection	118	Hospitalized and non-hospitalized patients.	38627	2.60%	The patients with coinfections were associated with increased odds of death and ICU admission.	CRD42020189763 Musuuza et al. (2021) (99)
Up to 3 September 2020	To evaluate the consequence of SARS-CoV-2 infection in patients with concurrent coinfections	20	Patients with coinfection with SARS-CoV-2 and other contemporary conditions.	205702	Cannot be evaluated	Patients with influenza had an increased risk of mortality during coinfection with SARS-CoV-2.	CRD420202064800 Sarkar et al. (2021) (96)
1 January 2020 to 31 December 2021	To evaluate the impact of coinfection with influenza or RSV on disease severity in COVID-19 patients	12	COVID-19 patients reporting any laboratory-confirmed coinfections with influenza or RSV	7862	Cannot be evaluated	Coinfection with influenza might be associated with a 2-fold increase in the risk for ICU admission and for mechanical ventilation among COVID-19 patients; no increase in the risk of death.	CRD42021283045 Cong et al. (2022) (97)
1 November 2019 to 13 August 2021	To evaluate community‐acquired viral coinfections and COVID‐19	59	SARS‐CoV‐2 mono and co‐infected patients	16643	1.54%	Patients coinfected with influenza were more likely to be dyspnoeic, and the odds of fatality, fever, and cough were similar.	CRD42021272235 Krumbein et al. (2023) (98)
1 January 2020 to 31 May 2023	To estimate the prevalence and severe clinical outcomes of influenza coinfection in COVID-19 patients.	95	COVID-19 patients.	62107	2.45%	Compared with mono-infected patients (COVID-19 only), the severe outcomes (including intensive care unit admission, mechanical ventilation support, and mortality) were significantly higher among patients coinfected with influenza A.	CRD42023423113 Yan et al. (2023) (100)

## 3 Co-administration of influenza virus and SARS-CoV-2 vaccines

During the COVID-19 pandemic, numerous studies documented co-infections of IAV and SARS-CoV-2 across diverse geographical regions (82, 83, 104). These dual infections were associated with significantly worse clinical outcomes, including increased disease severity, higher mortality rates, and greater strain on healthcare infrastructure (95–99). Concomitant vaccination against both COVID-19 and influenza can be used as a preventive measure.

### 3.1 Association between influenza vaccination and SARS-CoV-2 infection

**Figure 3:**
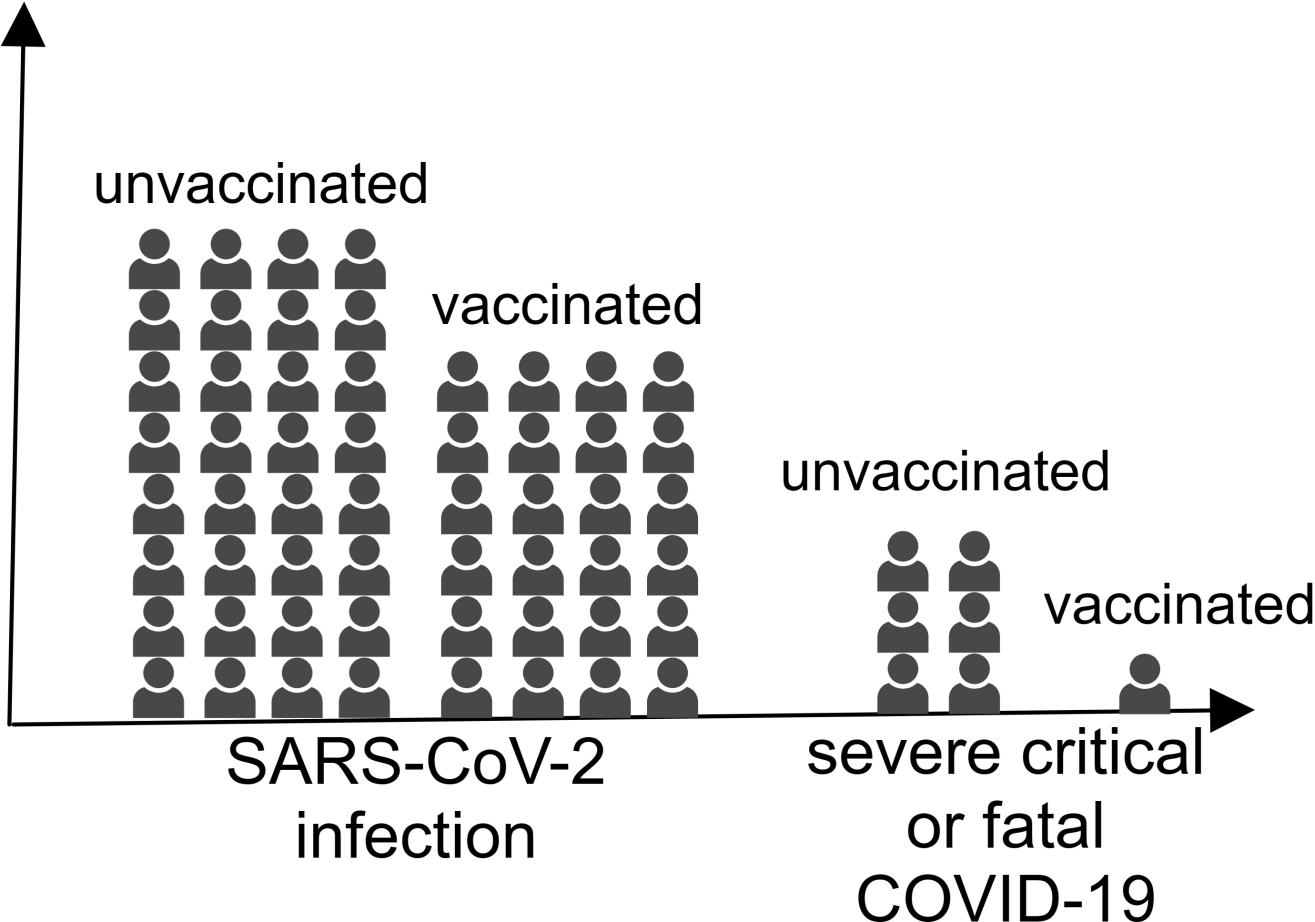
The individuals vaccinated with the influenza virus vaccine were less likely to be infected with SARS-CoV-2 and develop severe COVID-19.

Influenza vaccines are designed to protect from influenza virus infection, but growing evidence suggests they may also confer protective benefits against SARS-CoV-2 infection (105, 106). As early as October 2020, a systematic review conducted by Del Riccio et al. (2020) demonstrated significant inverse associations between influenza vaccination and COVID-19 severity outcomes, including reduced hospitalization rates and mortality among COVID-19 patients (**Figure 3**) (107).

Multiple epidemiological studies across diverse regions documented reduced SARS-CoV-2 infection rates among influenza-vaccinated populations (108–110). A striking example came from Italian healthcare workers, where influenza vaccination was associated with a 57% relative reduction in SARS-CoV-2 positivity (12.4% vs. 6.1%) compared to their unvaccinated counterparts (109). Comparable protective effects were observed among healthcare personnel in Qatar (29.7% effectiveness) (110) and Bahrain (54.3% effectiveness) (111). This protective association extends to high-risk populations, including elderly individuals, chronic disease patients, and healthcare workers (112, 113). A large-scale study of over 2 million seniors (eni years) revealed that influenza vaccination during the first 2 pandemic years was associated with a 22%–24% reduction in SARS-CoV-2 infection risk (114). Population-level evidence from meta-analyses encompassing 55,996,841 subjects further confirmed that influenza vaccination significantly reduced both SARS-CoV-2 infection rates and severe clinical outcomes (106). Notably, these observational findings were supported by randomized controlled trial data, demonstrating a 51% reduction in coronavirus infection risk among influenza vaccine recipients (115).

Beyond reducing SARS-CoV-2 infection rates, influenza vaccination demonstrated significant associations with mitigated disease severity in patients with COVID-19. Clinical studies revealed that vaccinated individuals experienced lower 60-day mortality rates and reduced ventilator requirements (116, 117). These protective effects were particularly pronounced in high-risk populations: among adults ≥66 years, influenza vaccination was associated with 17%–32% lower hospitalization rates and 27%–42% reduced mortality risk (114). Even among inpatients, unvaccinated hospitalized patients faced twice the ICU admission risk compared to their vaccinated counterparts, with enhanced protection observed in patients <65 years and non-obese individuals (118). Furthermore, influenza-vaccinated patients with COVID-19 exhibited decreased incidence of severe complications, including sepsis, stroke, and deep vein thrombosis, throughout disease progression (105). Current evidence strongly supports an inverse relationship between influenza vaccination and both SARS-CoV-2 infection risk and COVID-19 severity.

The protective mechanism of influenza vaccination against SARS-CoV-2 infection primarily involves the stimulation of innate immune defenses. One animal study demonstrated that prior influenza vaccination reduced viral shedding and mitigated pulmonary pathology upon SARS-CoV-2 challenge (119). This protection appears to be mediated through enhanced innate immune priming as the influenza vaccines activated key immune sentinels, including neutrophils, dendritic cells, and macrophages via Toll-like receptor (TLR) pathways, before viral exposure (120). Notably, TLR7-mediated recognition of SARS-CoV-2 may be particularly affected by this pre-activation (121). The vaccine-induced immune modulation extends to natural killer (NK) cells (122) and γδ T cells (123), with cross-reactive responses potentially attenuating SARS-CoV-2 pathogenicity (124). These innate immune cells that had been immunized generated a stronger targeted immune response that can be observed through epigenetic and metabolic changes after being exposed to immunostimulants (125). It is likely that interferons also played a role in this process after vaccination (126), while the MF59 adjuvant itself may provide non-specific protection when administered pre-exposure (127). The influenza vaccine primarily mediates protective immune responses against SARS-CoV-2 through non-specific immune mechanisms.

### 3.2 Co-administration of influenza and SARS-CoV-2 vaccination

As early as fall 2021, the WHO encouraged the simultaneous administration of the seasonal influenza vaccine and the SARS-CoV-2 vaccine at separate sites (128). Subsequently, acknowledging the compounded public health impact of concurrent influenza and COVID-19 threats, several nations, including the United Kingdom, adopted policies promoting dual vaccination (129). Thankfully, subsequent evidence has substantiated these policy decisions. Current clinical data now provide robust evidence for evaluating the outcomes of co-administration of influenza and SARS-CoV-2 vaccines.

According to the 2021 National Health Interview Survey in the US, a statistical study on COVID-19 infection status showed that the concurrent administration of both vaccines provided 35% infection protection, 6 percentage points higher than COVID-19 vaccination alone (130). A large-scale cohort study (n=600,000) demonstrated that dual influenza and COVID-19 vaccination was associated with a 27% reduced risk of SARS-CoV-2 infection and 45% lower all-cause mortality compared to COVID-19 vaccination alone (131). Experimental evidence from murine models corroborated these clinical findings, demonstrating that co-vaccination conferred complete protection against both SARS-CoV-2 and H1N1 challenge, with 100% survival maintenance and preservation of baseline body mass in all vaccinated subjects (91).

To systematically evaluate the safety and immunogenicity of concomitant vaccination, we conducted a PubMed search for clinical trials investigating coadministration of influenza and SARS-CoV-2 vaccines, identifying eight relevant studies published before 25 November 2024 (132–139) (**Table 2**). No significant safety issues regarding co-administration were reported. All age groups and all influenza and COVID-19 vaccines tested in the trials showed a high level of safety (132–136). Adverse events were usually mild in both the co-administration and mono-administration groups. Severe complications, while observed, exhibited low incidence rates with no significant differences compared to the control groups (134, 136). In all the included studies, co-administration of COVID-19 vaccines (including mRNA-1273, ChAdOx1, BNT162b2, CoronaVac, BBIBP-CorV, and NVX-CoV2373) and seasonal influenza vaccines (including HD-QIV, QIVc, QIVr, IIV4, and aTIV) had no significant negative impact on the immunogenicity of influenza or COVID-19 vaccines (132–136). Given the satisfactory immunogenicity and safety of coadministration of seasonal influenza vaccines and SARS-CoV-2 vaccines (140), public health institutions should play a central role in providing specific and tailored strategies.

**Table 2:** Safety and immunogenicity clinical trial studies on the co-administration of influenza and SARS-CoV-2 vaccinations.

Ref.	Walter et al. (2024) (137)	Izikson et al. (2022) (135, 138, 173)	Lazarus et al. (2021) (134)	Shenyu et al. (2022) (133)	Chen et al. (2022) (136)	Toback et al. (2022) (132)
Registration	ClinicalTrials.gov NCT05028361	ClinicalTrials.gov NCT04969276	EudraCT 2021–001124-18	ClinicalTrials.gov NCT04801888	ClinicalTrials.gov NCT04790851	ClinicalTrials.gov NCT04583995
Study dates	8 October 2021 to 14 June 2023	July/August 2021	April/June 2021	March/May 2021	10 to 15 March 2021	September/November 2020
Study design	Multicenter RCT	Multicenter RCT	Multicenter RCT	RCT	Multicenter RCT	Multicenter RCT
	(three centers)	(six centers)	(12 centers)		(three centers)	(33 centers)
Participants	335 persons:	306 adults:	679 adults:	480 adults:	1152 adults:	431 adults:
	≥5y, 100%	≥00 y, 100%	≥00 y, 33%	18–59 y, 100%	≥00 y, 50%	≥0% y, 7%
Vaccines	I, one flu vaccine: IIV4	I, one flu vaccine: HD-QIV	I, three flu vaccines: aTIV (with MF59C) QIVc (cellular) QIVr (recombinant)	I, one flu vaccine: IIV4	I, one flu vaccine: IIV4	I, two flu vaccines: aTIV, QIVc
	C, one COVID vaccine: BNT162b2	C, one COVID vaccine: mRNA-1273	C, two COVID vaccines: ChAdOx1 BNT162b2	C, one COVID vaccine: CoronaVac	C, one COVID vaccine: BBIBP-CorV	C, one COVID vaccine: NVX-CoV2373

RCT, randomized clinical trial; y, year; I, influenza vaccine; C, COVID-19 vaccine; aTIV, adjuvanted trivalent influenza vaccine; HD-QIV, high dose quadrivalent influenza vaccine; IIV4, quadrivalent split-virion inactivated influenza vaccine; QIVc, cellular quadrivalent influenza vaccine; QIVr, recombinant quadrivalent influenza vaccine.

## 4 Combination vaccines for SARS-CoV-2 and the influenza virus

**Figure 4:**
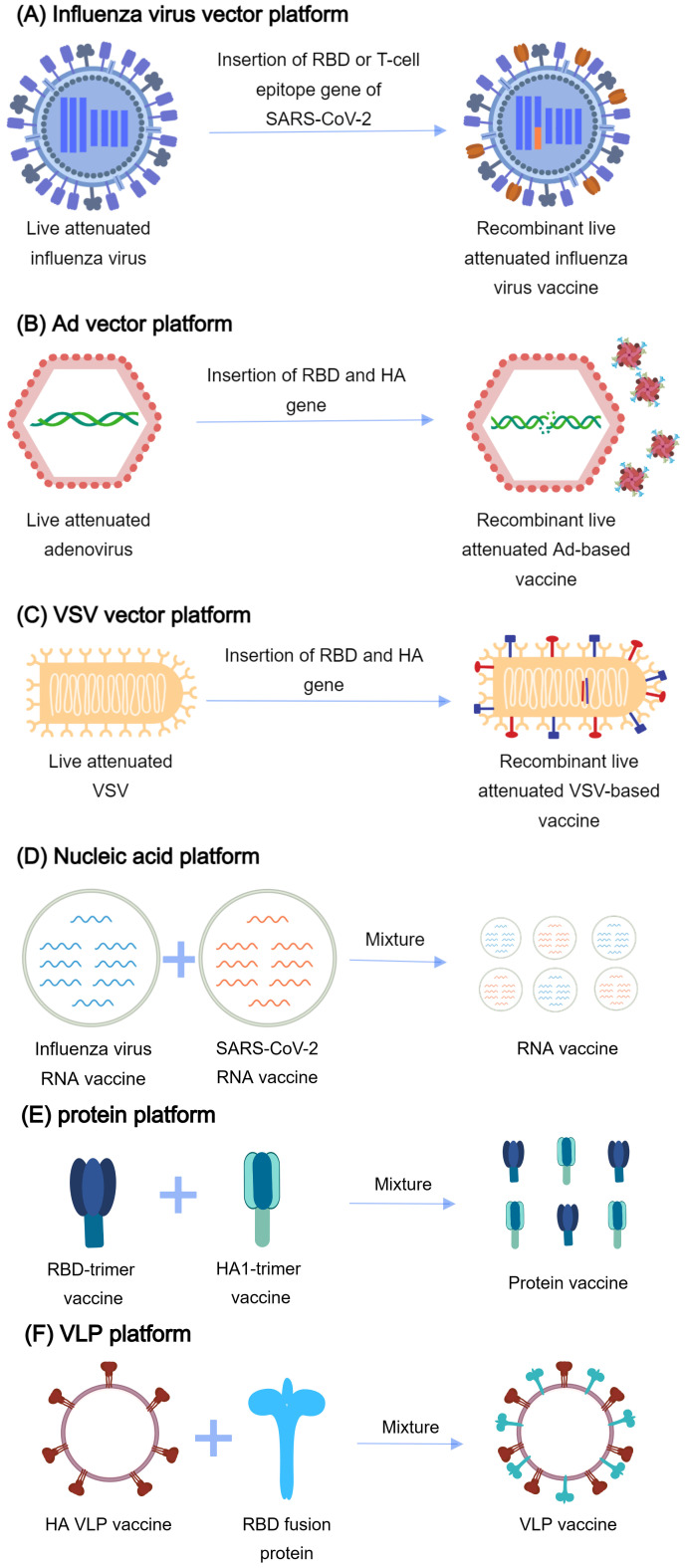
The platforms for vaccine development. **(A)** Influenza virus vector platform: The gene of RBD (receptor binding domain) or T-cell epitope of SARS-CoV-2 is inserted into the influenza virus genome. RBD or T-cell epitope is expressed on the surface of influenza virus particles. **(B)** Ad vector platform: The fusion gene of RBD of SARS-CoV-2, the stalk domain of hemagglutinin (HA) of influenza virus, and human ferritin is inserted into the adenovirus genome. The gene is delivered into the body by the recombinant adenovirus. The fusion immunogen self-assembles into nanoparticles. **(C)** VSV vector platform: The fusion gene of RBD of SARS-CoV-2 and Matrix Protein 2 Ectodomain (M2e) of the influenza virus are inserted into the vesicular stomatitis virus (VSV) genome. The gene is delivered into the body and expressed. **(D)** Nucleic acid platforms: A mixture of influenza virus RNA vaccine and SARS-CoV-2 RNA vaccine generates the combined vaccines. **(E)** Protein platforms: A mixture of the RBD-trimer vaccine and the HA1-trimer vaccine generates the combined vaccines. **(F)** VLP platform: Incorporation of GPI-RBD-GM-CSF (glycosylphosphatidylinositol, RBD, and granulocyte-macrophage colony-stimulating factor) fusion protein and virus-like particles (VLPs) generates the combined vaccines.

As of 25 November 2024, there were 27 successfully developed combination vaccine candidates employing diverse platform technologies documented in PubMed (**Figure 4**, **Table 3**). Among the developed combination vaccines, 12 utilized recombinant live-attenuated viral vectors, which included nine influenza virus vector vaccines, two adenovirus 68 (AdC68)-based vaccines (141, 142), and one recombinant vesicular stomatitis virus (rVSV)-based vaccine (143). In the design of influenza-vectored combination vaccines, SARS-CoV-2 antigenic sequences (e.g., RBD, spike protein, or T-cell epitopes) were inserted into the open reading frames (ORFs) of influenza viral proteins (e.g., NA or HA) in attenuated strains. This strategy enables dual antigen presentation, preserving influenza virion epitopes while expressing SARS-CoV-2 immunogens for robust immune recognition. To enhance immunogenicity, gene modifications and functional editing were employed to optimize fusion immunogen design. In contrast, AdC68 (141, 142) and rVSV (143) vector-based combination vaccines incorporated SARS-CoV-2 antigen sequences directly into the viral ORFs, leading to endogenous antigen expression in host cells. Unlike influenza vectors, which display antigens on virion surfaces, these systems presented antigens as fusion proteins, altering immune cell recognition mechanisms.

**Table 3:** Description of combination vaccines against SARS-CoV-2 and the influenza virus.

Vaccine candidate	Antigen	Platform	Does	Administration	Research phase	Reference
Recombinant rPR8-Hac/HEF-NBRBD vaccine	HEF RBD	Recombinant live attenuated vaccine	2	Intramuscular/Intranasal	Animal experiment	Zhao et al. (2022) (174)
AR-CoV/IAV vaccine	HA RBD	mRNA-based vaccine	2	Intramuscular	Animal experiment	Ye et al. (2022) (154)
RBD-trimer/HA1-trimer vaccine	HA1 RBD	Protein vaccine	2	Intramuscular	Animal experiment	Shi et al. (2022) (158)
Recombinant LAIV/SARS-CoV vaccine	Whole Influenza virus particle SARS-CoV-2-specific T-cell epitope	Recombinant live attenuated vaccine	2	Intranasal	Animal experiment	Isakova-Sivak et al. (2022) (175)
Recombinant TM-RBD-HA vaccine	Whole Influenza virus particle RBD	Recombinant live attenuated vaccine	2	Intranasal	Animal experiment	Chaparian et al. (2022) (176)
AdC68-CoV/Flu vaccine	Conserved stalk of HA(H7N9) RBD	Recombinant adenovirus-based vaccine	2	Intramuscular	Animal experiment	Cao et al. (2022) (141)
VLP-RBD-GM-CSF-IL-12 hybrid vaccine	HA RBD	Fusion protein VLP vaccine	2	Subcutaneous/Intramuscular	Animal experiment	Bommireddy et al. (2022) (152)
qNIV/CoV2373	Full-length HA Spike protein	Nanoparticle vaccine	2	Intramuscular	Phase I, II, III (NCT05519839, NCT04961541)	Massare et al. (2021) (157) (2024) (165)
dNS1-RBD	Whole Influenza virus particle RBD	Recombinant live attenuated vaccine	2	Intranasal	Animal experiment Phase I, II (ChiCTR2000037782, ChiCTR2000039715, ChiCTR2100048316)	Chen et al. (2022) (177) Zhang et al. (2023) (178) Zhu et al. (2022) (164)
Flu-RBD	Whole Influenza virus particle RBD	Inactivated vaccines	2	Intramuscular	Animal experiment	Wang et al. (2023) (179)
DelNS1-RBD4N-DAF	Whole Influenza virus particle RBD	Recombinant live attenuated vaccine	2	Intranasal	Animal experiment	Deng et al. (2023) (180)
H1Delta	HA stalk(H1N1) RBD	Recombinant protein vaccines	3	Intramuscular	Animal experiment	Li et al. (2023) (159)
v-EM2/SPΔC1_Delta_	M2e Spike protein	Recombinant rVSV-based vaccine	2	Intramuscular/Intranasal/Oral	Animal experiment	Ouyang et al. (2023) (151) Ao et al. (2022) (143)
PR8NARBD/WSN	Whole Influenza virus particle RBD	Recombinant live attenuated vaccine	1	Intranasal	Animal experiment	Wang et al. (2023) (181)
mRNA-1073	Full-length HA Spike protein	mRNA vaccines	1	Intramuscular	Phase I, II	NCT05375838
mRNA-1083	Full-length HA RBD	mRNA vaccines	1	Intramuscular	Phase I, II, III	NCT06508320 NCT06097273 NCT05827926
FLU-COVID	Full-length HA Spike protein	Recombinant protein vaccines	3	Intramuscular	Animal experiment	Huang et al. (2024) (160)
Co/Flu WPV	Whole Influenza virus particle Whole SARS-CoV-2 virus particle	Inactivated vaccines	1	Intramuscular	Animal experiment	Handabile et al. (2024) (163)
HA+ Omicron S	Full-length HA Spike protein	Recombinant protein vaccines	2	Intramuscular	Animal experiment	Zhang et al. (2024) (161)
M2SR SARS-CoV-2	Whole Influenza virus particle RBD	single-replication virus vector vaccine	2	Intranasal	Animal experiment	Hill-Batorski et al. (2024) (150) Moser et al. (2023) (149)
AdC68-HATRBD	HA(H1N1) RBD	Recombinant adenovirus-based vaccine	2	Intranasal	Animal experiment	Xing et al. (2024) (142)
LAIV/CoV-2	Whole Influenza virus particle Spike protein	Recombinant live attenuated vaccine	2	Intranasal	Animal experiment	Stepanova et al. (2024) (182)
LAIV-RBD	Whole Influenza virus particle RBD	Recombinant live attenuated vaccine	2	Intranasal	Animal experiment	Stepanova et al. (2024) (183)
FLUCOV-10	HA Spike protein	mRNA vaccine	2	Intramuscular	Animal experiment	Wang et al. (2024) (155)
H1Delta mRNA	H1 RBD	mRNA vaccine	2	Intramuscular	Animal experiment	Hao et al. (2024) (156)
S/H1/N1 VLPs	HA and NA(H1N1) Spike protein	VLPs vaccine	2	Intramuscular	Animal experiment	Sanchez-Martinez et al. (2024) (153)
rTTV-RBD-HA2	Conserved stalk region of HA (pdmH1N1 and nH7N9) RBD (SARS-CoV-2, SARS-CoV, and MERS-CoV)	Fusion protein	3	Intramuscular/Intranasal	Animal experiment	Gao et al. (2024) (162)

Among existing combination vaccine platforms, viral vectors—particularly influenza-based systems—represented the predominant strategy. Leveraging well-established live-attenuated influenza vaccine (LAIV) technology, these vectors effectively induced T-cell immunity (144) and have been successfully engineered to elicit robust responses against incorporated SARS-CoV-2 epitopes (145, 146). However, pre-existing influenza immunity may attenuate their efficacy, prompting the use of alternative vectors such as AdC68 and rVSV (147, 148). To address potential virulence reversion risks in LAIV platforms, single-replication influenza vectors (e.g., M2SR SARS-CoV-2) offered enhanced safety (149, 150). Viral vector vaccines inherently mimic natural infection, enabling potent immunogenicity even without adjuvants. Their compatibility with mucosal delivery [e.g., intranasal/oral administration (151)] further enhanced mucosal immunity—a critical advantage over conventional platforms.

Among the developed combination vaccine candidates, two utilized VLP technology, incorporating fusion antigens from both influenza and SARS-CoV-2 into self-assembling VLPs (152, 153). Other platforms included mRNA vaccines (154–156), nanoparticle vaccines (157), and recombinant protein vaccines (158–162). These were typically developed through physical mixing of validated monovalent vaccines. The latter approach enabled quicker clinical translation, as pre-approved monovalent components already had established safety and efficacy profiles. Notably, only one inactivated vaccine candidate was developed (163), created by mixing two separate inactivated vaccines. This limited representation suggests that traditional inactivated vaccine strategies may be less suitable for developing broad-spectrum, multivalent vaccines compared to more modular platforms.

All combination vaccine candidates demonstrated the capacity to elicit robust neutralizing antibodies and confer dual protection against influenza and SARS-CoV-2 infections. However, their immunological profiles exhibited significant variations in the durability of antibodies, the strength of cellular and humoral immunity, the presence or absence of mucosal immunity, and the level of protection against different viral subtypes. These functional differences stem primarily from divergent epitope selection strategies for influenza (e.g., HA/NA variants) and SARS-CoV-2 (e.g., RBD/spike variants) antigens across platforms. The modular design and manufacturing flexibility of these bivalent systems (VLP, mRNA, nanoparticle, etc.) are accelerating the development of next-generation multivalent vaccines with broader spectrum coverage.

Four vaccine platforms had advanced to clinical trials: two RNA vaccines (NCT05375838, NCT06097273), one influenza virus vector vaccine (ChiCTR2000037782, ChiCTR2000039715, ChiCTR2100048316) (164), and one nanoparticle vaccine (NCT05519839, NCT04961541) (165). All the platforms had demonstrated acceptable safety profiles in early-phase trials. mRNA-1073, mRNA-1083, and qNIV/CoV2373 were developed by monovalent vaccines that had been validated in clinical trials. While preliminary clinical data support the safety profiles of these four vaccine candidates, their efficacy in human populations remains to be established through large-scale Phase III trials.

Despite the proven efficacy of combination vaccines against both influenza and COVID-19, vaccine hesitancy remains a significant challenge in immunization campaigns. Multiple factors contribute to vaccination reluctance, including a limited understanding of communicable diseases (166), insufficient awareness of vaccine importance (167), socioeconomic disparities, and variable public health infrastructure (168). First and foremost, the government must ensure the safety of vaccines, as this is a matter of paramount importance (169). Educational interventions can enhance public awareness regarding infections and vaccines, thereby increasing individuals’ willingness to undergo vaccination (170). Furthermore, offering vaccination services in public venues such as pharmacies not only provides greater convenience but also has the potential to boost vaccination rates (171). In a questionnaire survey, it was found that compared with receiving the flu vaccine and the COVID-19 vaccine alone, the majority of American consumers and healthcare providers may be more inclined to receive a single-dose flu + COVID combined vaccine, an advantage of a combined vaccine (172).

## 5 Conclusion and future prospects

During the COVID-19 pandemic, influenza cases remained at low levels due to implemented containment measures. However, following the conclusion of COVID-19 restrictions, influenza incidence has rebounded to pre-pandemic levels. The decline in COVID-19 case numbers and the WHO’s declaration that COVID-19 no longer constitutes a Public Health Emergency of International Concern (PHEIC) have inadvertently fostered public misperceptions that SARS-CoV-2 has been eradicated. Unfortunately, the statement only means that COVID-19 is no longer an emergency and no longer requires international coordination. COVID-19 is also a persistent health issue. According to the 2023–2025 COVID-19 Strategic Preparedness and Response Plan released by WHO, millions of people each week continue to be confirmed as infected/reinfected and thousands of people are dying around the world. Given the ongoing uncertainties posed by the potential evolution of SARS-CoV-2, WHO committee members emphasized during the deliberative session that it is time to transition to long-term management rather than neglect ongoing surveillance despite the significant reduction in COVID-19 severity.

Over the past 3 years, social distancing and isolation measures proved to be the most effective but ultimately unsustainable strategy for containing viral transmission, as they significantly disrupted socioeconomic functioning. Against both SARS-CoV-2 and influenza, vaccines remain the most viable solution for interrupting transmission chains. To date, over 13 billion doses of COVID-19 vaccines have been administered globally. According to an Imperial College London study on excess mortality (2022), emergency-authorized vaccines saved an estimated 20 million lives between December 2020 and December 2021 alone. As previously discussed, co-administration of influenza and COVID-19 vaccines provided superior protection compared to monovalent vaccination. Animal studies demonstrated that combination vaccines not only enhanced efficacy but also reduced immunization frequency and adverse events. Although human clinical validation is ongoing, this approach represents a promising strategy for pandemic containment. New mutated strains and immune escape drive scientists to adopt new technologies and platforms in constructing more effective and broad-spectrum combination vaccines.

The combination vaccines mentioned above can provide protection against a variety of subtypes of influenza viruses and SARS-CoV-2. However, the range of virus subtypes that vaccines can target is restricted, and their efficacy against newly emerging subtypes cannot be assured. It is imperative to allocate additional resources toward vaccine research and development. Furthermore, public health authorities should enhance promotional efforts to raise public awareness regarding the significance of vaccination and encourage greater acceptance. Simultaneously, measures should be implemented to improve the accessibility and convenience of vaccination services.

Combination vaccines offer governments significant cost-containment advantages through streamlined production and distribution, while simultaneously improving population-level vaccine uptake by reducing required immunization visits. Combination vaccine design platforms provide valuable foundational experience for future vaccine development. The focus of our discussion in this article is combination vaccines for influenza and COVID-19, but the vaccine platform can be used for any two or more pathogens, even cancer. Even if efforts to develop new combination vaccine technologies and platforms do not yield direct benefits against COVID-19 and influenza, they could still contribute to combating other diseases, like malignant tumors or other infectious diseases, or even a new pandemic. The flexibility and scalability of the vaccine strategies mentioned above provide more opportunities for experimentation in vaccines and greater potential for creating broad-spectrum, multivalent vaccines. Although there is no vaccine that can simultaneously protect people against annual seasonal influenza and SARS-CoV-2 variants, it may be on the way.
